# Facile Fabrication of Nanofibrillated Chitin/Ag_2_O Heterostructured Aerogels with High Iodine Capture Efficiency

**DOI:** 10.1038/s41598-017-04436-8

**Published:** 2017-06-27

**Authors:** Runan Gao, Yun Lu, Shaoliang Xiao, Jian Li

**Affiliations:** 1 Material Science and Engineering College, Northeast Forestry University, Harbin, 150040 P.R. China; 2 Research Institute of Wood Industry, Chinese Academy of Forestry, Beijing, 100091 P.R. China; 3 Key Laboratory of Bio-based Material Science and Technology Ministry of Education, Northeast Forestry University, Harbin, 150040 P.R. China

## Abstract

Nanofibrillated chitin/Ag_2_O aerogels were fabricated for radioiodine removal. Chitin was first fabricated into nanofibers with abundant acetyl amino groups (–NHCOCH_3_) on the surface. Then, highly porous chitin nanofiber (ChNF) aerogels were obtained via freeze-drying. The ChNF aerogels exhibited a low bulk density of 2.19 mg/cm^3^ and a high specific surface area of 179.71 m^2^/g. Ag_2_O nanoparticles were evenly anchored on the surfaces of ChNF scaffolds via strong interactions with –NHCOCH_3_ groups, subsequently yielding Ag_2_O@ChNF heterostructured aerogels. The composites were used as efficient absorbents to remove radioiodine anions from water and capture a high amount of I_2_ vapor in the forms of AgI and iodine molecules. The adsorption capacity of the composite monoliths can reach up to 2.81 mmol/g of I^−^ anions. The high adsorbability of the composite monolithic aerogel signifies its potential applications in radioactive waste disposal.

## Introduction

Aerogels are low-density solid materials and are characterized by a highly accessible mesoporous network that is composed of three-dimensionally interconnected particles^1^. Aerogels integrate unusual properties, such as low density, high specific surface area, low heat conductivity, and high transparency, that are individually present in other materials. Given these outstanding characteristics, aerogels have been utilized as Cherenkov detectors, catalyst support media, and absorbers^2, 3^. Organic aerogels appeared as a compelling alternative to inorganic aerogels after Pekala *et al*.^4^ conducted fundamental research on phenolic-resin-based aerogels. Resorcinol/formaldehyde aerogel is the first developed and the most investigated organic aerogel^5^. Many other reactant systems, such as melamine–formaldehyde^6^, resorcinol–furfural^7^, cresol–formaldehyde^8^, phenol–furfural^9^, polyurethane^10^, polyisocyanate, and polyolefin^11, 12^, have been developed in later works by other researchers. Recently, cellulose or chitin have received increased attention as aerogel building blocks because of their outstanding high stiffness, low density, high aspect ratio, and large specific surface area^13–17^. Numerous reports on nanofabrillated-cellulose-based aerogels have been widely published^18^. Similar studies on chitin aerogels, however, have just come into force. Yun Lu *et al*. successfully fabricated chitin nanofibers for assembly into spongy foam^19^. Bo Duan *et al*. reported the use of chitin sponge materials for oil–water separation^20^. Chitin-based aerogels have also been developed and reported as efficient base catalysts^21^.

Ag_2_O has received intensive research attention because of its high reactivity with I to form insoluble AgI. This Ag_2_O characteristic can be applied in radioactive iodine waste disposal. Among a variety of harmful radioactive isotopes derived from the fission of uranium 235, I^129^ and I^131^ are major factors that increase morbidity rate. Directly immobilizing radioactive iodine with Ag_2_O, however, is unfeasible as removal capacity and dynamics depend on the specific surface area of Ag_2_O. Although nanosized Ag_2_O particles with large specific surface areas have high capacity for radioiodine capture, the separation and recovery of the nanoparticles remain problematic. A leading research study has anchored Ag_2_O nanoparticles on titanate-based nano-adsorbents and indicated that the key countermeasure to prevent agglomeration is the even dispersion of fine inorganic nanoparticle granules on a carrier with a high specific area^22^.

In the present work, we introduced porous ChNF aerogels as supports for Ag_2_O-nanoparticle decoration to develop a high-efficiency, sustainable absorbent. One-dimensional (1D) nanofibers were isolated from chitin, an environmentally friendly bio-resource with superior mechanical properties, such as strength and flexibility, which are comparable with those of collagen. These properties make ChNF a favorable scaffold for inorganic nanoparticle decoration. Specific acetyl amino groups (–NHCOCH_3_) on ChNF scaffolds facilitate the intensive chelation of Ag^+^ on the ChNF surface through the donation of a lone electron pair by acetyl amino groups (–NHCOCH_3_)^23^. Hydroxyl groups on fibers also provide a unique platform for surface modification. After preparation into ChNF aerogels, the 3D network of entangled nanofibers provides a large specific area for the even dispersion of fine Ag_2_O nanoparticles without agglomeration. When used as adsorbents, numerous mesopores allow the access of radioactive ions in water to Ag_2_O nanoparticles, thus guaranteeing the high adsorbability of radioactive iodine anions.

To develop a high-efficiency iodine absorbent, we prepared Ag_2_O-anchored ChNF (Ag_2_O@ChNF) heterostructured aerogel via a facile process. First, we prepared ChNF nanofiber aerogels through chemical pretreatment combined with high-intensity ultrasonication and freeze-drying. We then isolated purified chitin into nanofibers with individual diameters of approximately 50 to 300 nm. We replaced water with *tert*-butanol to induce the crosslinking of chitin nanofibers to form gels. Finally, we obtained monolithic ChNF aerogels through freeze-drying. This monolithic material showed low bulk density and high specific surface area. Ag_2_O nanoparticles were anchored on chitin nanofibers via simple chemical reactions in solution. The size of Ag_2_O nanoparticles could be controlled by adjusting the initial concentration of ammoniated silver solution (Tollen’s reagent). Ultrasonication treatment was also introduced to prevent the agglomeration of nanoparticles. The Ag_2_O-loaded monoliths efficiently remove I^−^ from water and capture a significant amount of I_2_ vapor for effective fixation in the forms of AgI and iodine molecules.

## Results and Discussion

**Figure 1:**
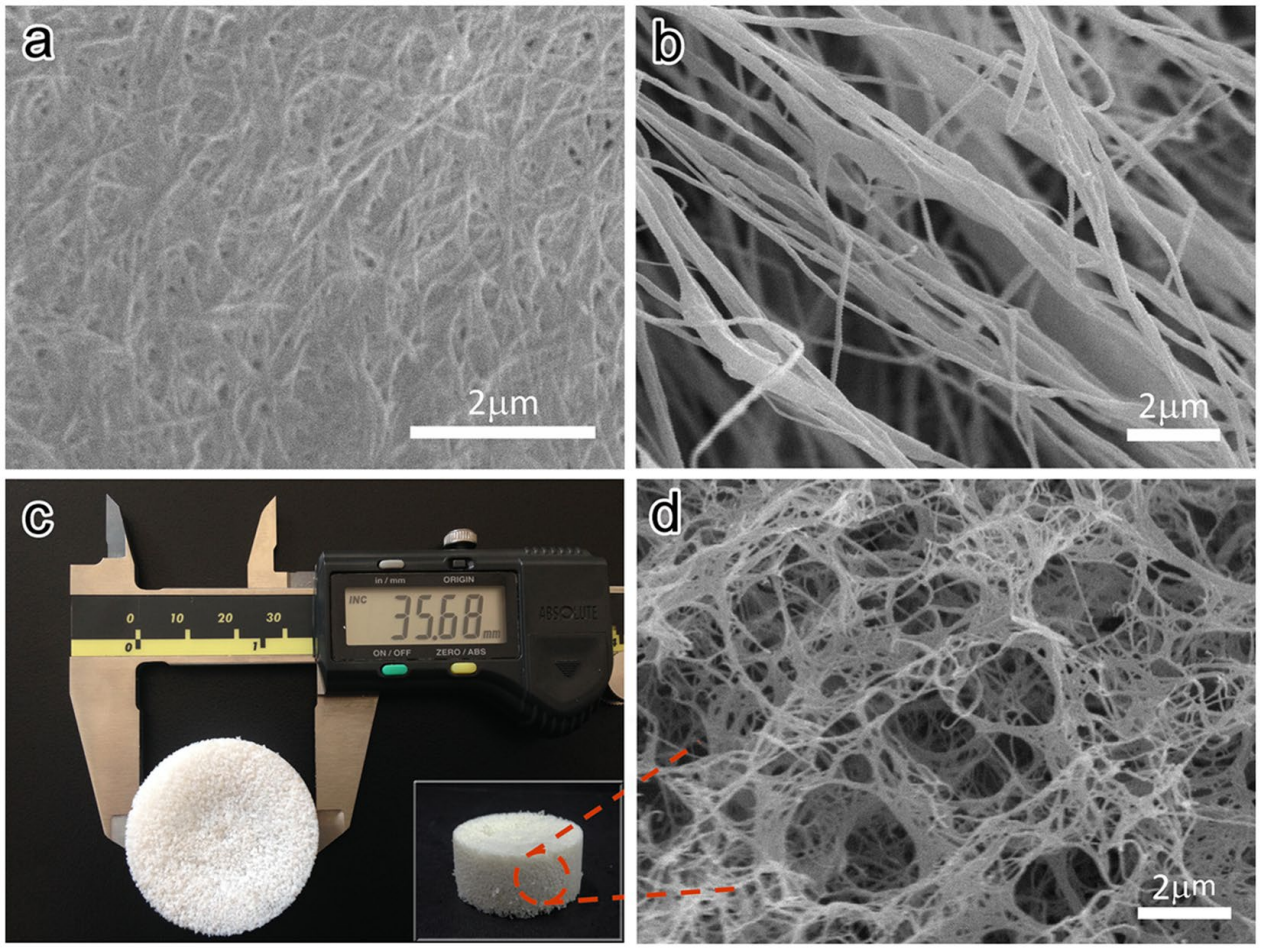
SEM images of (**a**) chitin after chemical pretreatment and (**b**) ChNFs after 30 min of ultrasonication. (**c**) Macrogragh of the ChNF aerogel. (**d**) Microscopic structure of the ChNF aerogel.

Acid pretreatment partially deacetylated ChNFs. The deacetylation degree (DD) of ChNFs was 32.8%, as evaluated from ^13^C solid-state NMR (see Supplementary Fig. S1). Chemical pretreatments slackened tightly bonded chitin fibril bundles in addition to eliminating proteins, mineral salts, and lipids from chitin samples. The microstructure of the chemically purified chitin is presented in Fig. 1(a). After pretreatment, interlaced chitin fibril bundles are clearly visible in the scanning electron microscopy (SEM) image. Subsequently, to obtain ChNFs, high-intensity ultrasonic treatment was applied. During ultrasonication, an active zone with a high concentration of cavities was created. The sonication probe of the ultrasonicator worked as an energy transfer device that vibrated the liquid to form hollow bubbles, or cavities^24–26^. Cavitation gradually disassembled fibril bundles into nanofibers because the intensive collapse of cavities on the surfaces of the fibril bundles effectively split fibers along the axial direction. Thus, the final nanofibers were obtained. Although ultrasonication treatment can avoid the use of chemicals and has a smaller impact on the environment, it can broaden the diameter distribution of nanofibers. Figure 1(b) presents the morphology of ChNFs after 30 min of ultrasonication. The figure shows long, straight individual nanofibers with individual diameters of 50 to 300 nm. The mean diameter of ChNFs was 153 nm. More detailed information about the morphology and diameter distribution of ChNFs is presented in Supplementary Fig. S2. The individual nanofibers were then assembled into ultralight aerogels. After replacing the water in the sonication suspension with *tert*-butanol, the viscosity of the suspension increased due to the entanglement of ChNFs and a pulpy gel body was obtained (see Supplementary Fig. S3). After freeze-drying, monolithic ChNF aerogels were obtained, as shown in Fig. 1(c). The ChNF aerogels that were prepared in the present study had a very low density of approximately 2.19 mg/cm^3^. This monolithic material did not obviously shrink during freeze-drying. Moreover, its shape corresponded with the shape of the cylindrical plastic molds, indicating the absence of structural collapse. Figure 1(d) shows the microstructure of the ChNF aerogels. The three-dimensionally interconnected nanofiber skeleton of nanofibrillated chitin is clearly visible in the figure.

N_2_ adsorption–desorption under liquid nitrogen flow provides valuable structural information about porous materials. ChNF aerogels have a large specific surface area of 179.71 m^2^/g and high porosity of 99.85%. The average pore diameter and the total pore volume of ChNF aerogels were 16.37 nm and 0.76 cm^3^/g, respectively. The porosity of aerogels was calculated by

1 $${P}_{a}=(1-\frac{{\rho }_{a}}{{\rho }_{c}})\times 100$$

where *ρ*_a_ is the density of the ChNF aerogel and *ρ*_c_ is the density of bulk chitin (1.425 g/cm^3^)^27^.

**Figure 2:**
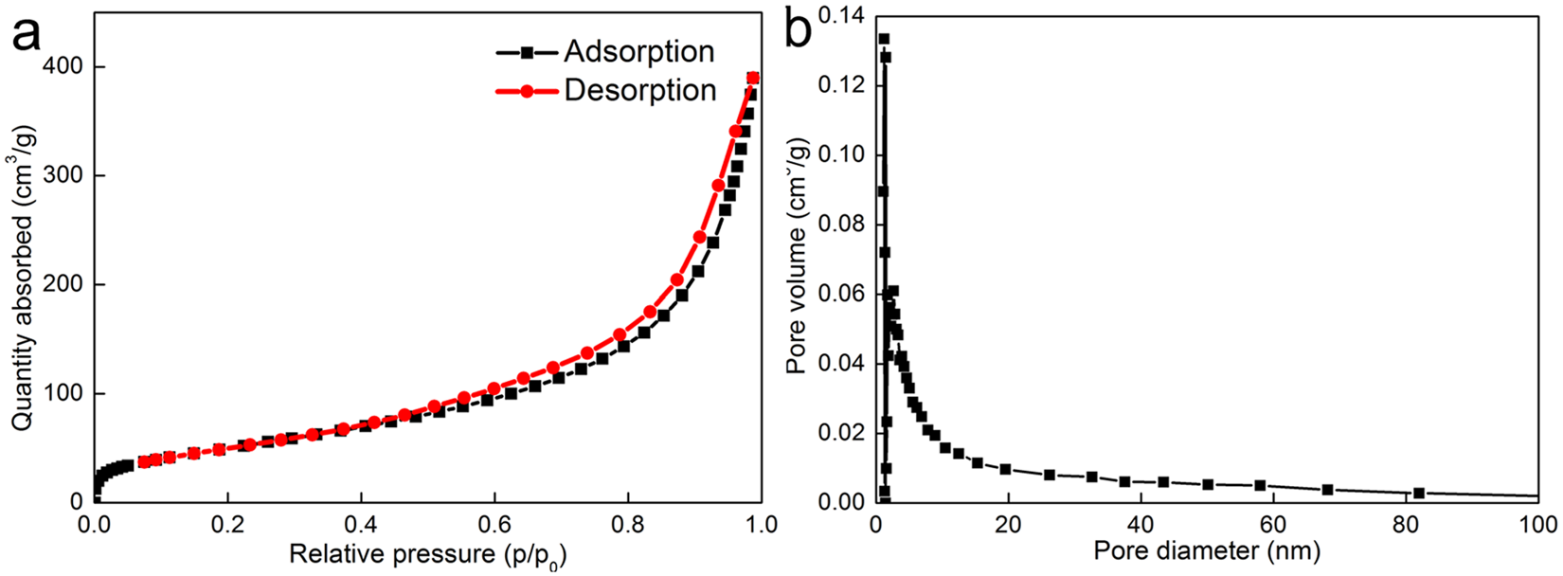
Adsorption–desorption isotherms (**a**) and pore size distribution (**b**) of ChNF aerogels.

Figure 2(a) and (b) show the nitrogen adsorption–desorption isotherms and Barrett–Joyner–Halenda (BJH) pore size distribution of ChNF aerogels. According to IUPAC classification, ChNF aerogels exhibited type IV adsorption isotherms. The adsorption branch nearly overlapped with the desorption branch when P/P_0_ was less than 0.4. The presence of mesopores was demonstrated when a type H3 hysteresis loop appeared when P/P_0_ exceeded 0.4^28^. The main pore diameter distribution of ChNF aerogels was 2–20 nm, as shown in Fig. 2(b), which further confirmed the mesoporous structure of the samples.

Ag_2_O@ChNFs with different Ag_2_O loading concentrations were investigated via N_2_ adsorption–desorption. The bulk density, specific surface area, and pore volume of pristine and modified aerogels are listed in Table 1. Specific surface area and pore volume gradually decreased as Ag_2_O concentration increased. This behavior indicated that porosity decreases in accordance with the thickening of the ChNF scaffold after anchoring inorganic nanoparticles.

**Table 1:** Bulk densities, specific surface areas, and porosities of pristine and Ag_2_O-anchored aerogels.

Ag_2_O (wt%)	*ρ*_aerogel_ (mg/cm^3^)	BET specific surface area (m^2^/g)	Pore volume (cm^3^/g)
0	2.19	171.91	0.76
31	4.20	131.96	0.66
56	4.46	92.58	0.33
122	5.31	74.31	0.27

Ag_2_O@ChNF aerogels were obtained after immersing ChNF networks in ammoniated silver solution followed by freeze-drying. Prior to freezing, the samples were treated with ultrasonication again to ensure homogeneous dispersion and to prevent the agglomeration of fine nano-granules. Ag_2_O nanoparticles formed and anchored on the ChNF network through following reactions:

1 $${{\rm{AgNO}}}_{3}+2{{\rm{NH}}}_{3}\times {{\rm{H}}}_{2}{\rm{O}}\to {\rm{Ag}}{({{\rm{NH}}}_{3})}_{2}^{+}+{{\rm{NO}}}_{3}^{-}+2{{\rm{H}}}_{2}{\rm{O}}$$

2 $${\rm{Ag}}{({{\rm{NH}}}_{3})}_{2}^{+}+{\rm{NaOH}}\to {\rm{AgOH}}+2{{\rm{NH}}}_{3}+{{\rm{Na}}}^{+}$$

3 $$2{\rm{AgOH}}\to {{\rm{Ag}}}_{2}{\rm{O}}+{{\rm{H}}}_{2}{\rm{O}}$$

**Figure 3:**
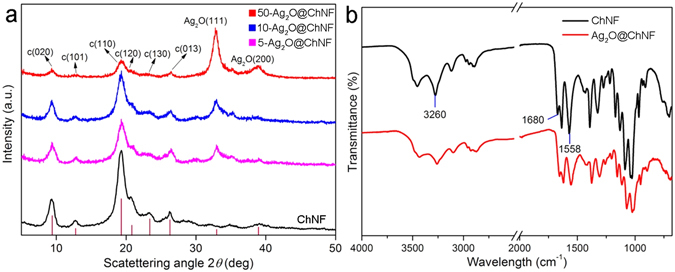
(**a**) XRD spectrogram of ChNF and Ag_2_O@ChNF aerogels with different loading concentrations. (**b**) FTIR spectrogram of ChNF and Ag_2_O@ChNF.

X-ray diffraction (XRD) and FTIR analyses provided evidence for the formation of Ag_2_O nanoparticles via the above reactions. XRD data are presented in Fig. 3(a). In this figure, the spectral line of pristine monolithic ChNF materials is at the bottom and the spectra of aerogels with different loading concentrations are listed upward in succession. The diffraction peaks of monolithic ChNF aerogels appeared at 9.4°, 12.8°, 19.3°, 20.5°, 23.5°, and 26.3°, which corresponded to the planes of (020), (101), (110), (120), (130), and (013), respectively. The crystallinity of ChNFs was 84.1% and was estimated by crystalline index (CrI;%) expressed as CrI_110_ = (*I*_110_−*I*_am_) × 100/*I*_110_. Characteristic Ag_2_O peaks appeared after the precipitation of precursors on ChNF scaffolds. The peaks at 32.8° and 38.1° corresponded to the (111) and (200) planes of Ag_2_O. Ag_2_O nanoparticles covered the ChNF network as the concentration of starting ammoniated silver solution increased. This process was indicated in the XRD pattern as the gradual decay or even the overlapping of characteristic chitin peaks.

The interaction between Ag_2_O nanoparticles and ChNF scaffolds was further investigated via FTIR analysis, as shown in Fig. 3(b). For ChNF, the peak at the frequency of 1680 cm^−1^ was characterized as C = O stretching, which proved the presence of acetyl amino groups. After loading, a negative shift to 1660 cm^−1^ occurred. In addition, a characteristic peak at 1558 cm^−1^ that was assigned to C–N stretching and N–H deformation broadened and decreased. This pattern indicated a strong interaction between acetyl amino groups and Ag^+^. The adsorption peak at 3260 cm^−1^ was attributed to the stretching vibrations of intermolecular hydrogen bonds in chitin, where C(6)OH groups are hydrogen-bonded to N–H in adjacent molecular chains. After Ag_2_O deposition, a negative shift to 3244 cm^−1^ occurred and the peak was broadened. This result demonstrated that hydrogen bonds formed between the hydroxyl groups of chitin and Ag_2_O nanoparticles, which increased the attraction of Ag^+^ to ChNF scaffolds.

**Figure 4:**
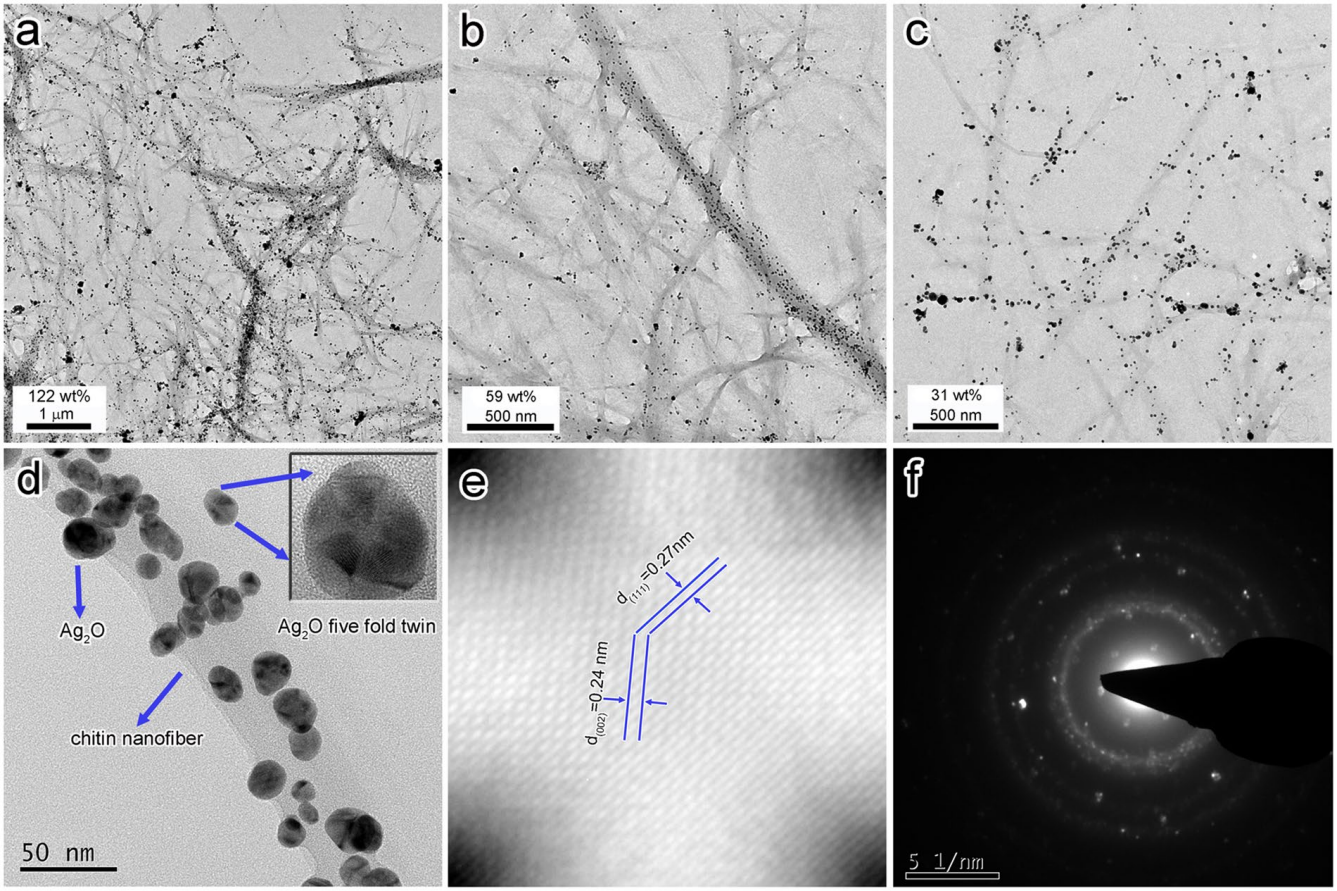
TEM images of (**a**) 50-Ag_2_O@ChNF, (**b**) 10-Ag_2_O@ChNF, and (**c**) 5-Ag_2_O@ChNF. (**d**) HRTEM image of 50-Ag_2_O@ChNF, inset: the five-fold twinning particle of Ag_2_O. (**e**) Inverse FFT (IFFT) image-scaled lattice fringe of Ag_2_O (111) plane and (002) plane. (**f**) SEAD image of Ag_2_O.

Characterization via transmission electron microscopy (TEM) provided additional structural information about Ag_2_O@ChNF aerogels. Figure 4(a–c) shows the TEM images of Ag_2_O@ChNF samples with different loading concentrations. In 50-Ag_2_O@ChNF, the sample with the highest loading concentration (the labeling method of samples is detailed in the experimental section), nanoparticles were densely and evenly anchored on ChNF networks with negligible agglomeration. By contrast, smaller nanoparticles were sparsely distributed and scattered on 10-Ag_2_O@ChNF and 5-Ag_2_O@ChNF. The loading content in the final product could be controlled to ~122 wt% (50-Ag_2_O@ChNF), ~56 wt% (10-Ag_2_O@ChNF), and ~31 wt% (5- Ag_2_O@ChNF). Furthermore, the size of Ag_2_O nanoparticles could be controlled by the initial concentration of Tollen’s reagent. Particle sizes were then measured and recorded (Supplementary Fig. S4). As the concentration of Tollen’s reagent increased, the mean diameter of Ag_2_O nanoparticles increased from 8 nm in 5-Ag_2_O@ChNF, to 13 nm in 10-Ag_2_O@ChNF, and finally to 16 nm in 50-Ag_2_O@ChNF. These results were in a good agreement with results that were calculated using the Scherrer equation (See Supplementary Table S2). The HRTEM image of Ag_2_O@ChNF is presented in Fig. 4(d). Nanoparticles were evenly distributed on ChNF scaffolds without agglomeration. High surface energies make nanoparticles more likely to aggregate. Ultrasonication with the appropriate handling time, however, can effectively alleviate aggregation. Before the standing process for precursor precipitation, the samples were treated with ultrasonication for 5 min. During ultrasonication, the explosion of cavities exerted shear force on nanoparticles. Shear force breaks down Van der Waals attraction and prevents agglomeration. The inset in Fig. 4(d) shows that the Ag_2_O phase has a face-centered cubic structure. This particle formed a five-fold twinning feature that is common to the cubic structural phase. Figure 4(e) provides information about lattice fringes of Ag_2_O nanoparticles. The lattice spacing of plane (002) was 0.24 nm and that of plane (111) was 0.27 nm. The SAED image showed a series of intermittent diffraction rings that originated from the random orientations of the Ag_2_O nanoparticles.

### Iodine adsorption

**Figure 5:**
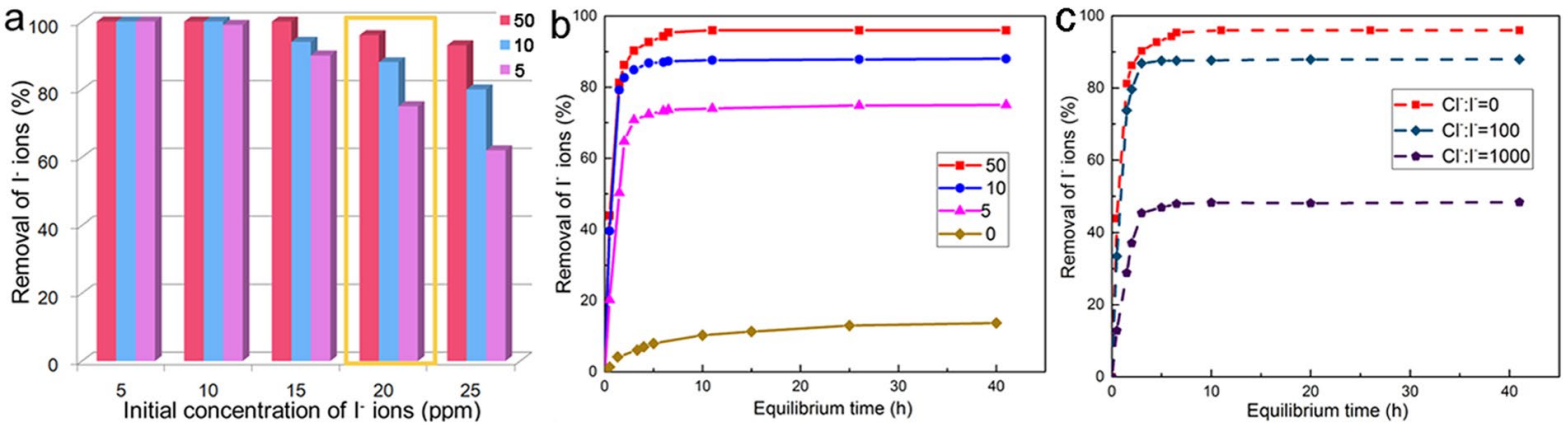
(**a**) Removal of I^−^ anions at different concentrations by 50-Ag_2_O@ChNF, 10-Ag_2_O@ChNF, and 5-Ag_2_O@ChNF; (**b**) The kinetic adsorption curve of I^−^ anions by Ag_2_O@ChNF composites and ChNF aerogel; (**c**) I^−^ adsorption kinetics with 20 ppm I^−^ anions with different concentration of Cl^−^ as a competitive anion.

As shown in Fig. 5(a), below 20 ppm NaI, all adsorbents immobilized more than 80% of I^−^, whereas 50-Ag_2_O@ChNF removed 100% of I^−^. The kinetic isotherms of I^−^ adsorption at 20 ppm I^−^ are presented in Fig. 5(b). During the first 7 h of adsorption at NaI concentrations of 20 ppm or less, 50-Ag_2_O@ChNF, 10-Ag_2_O@ChNF, and 5-Ag_2_O@ChNF reached saturated sorption capacity, which is equivalent to adsorption quantity at 48 h. The adsorption capacities of the samples showed the following order: 50-Ag_2_O@ChNF, ~2.81 mmol/g^−1^; 10-Ag_2_O@ChNF, ~2.72 mmol·g^−1^; and 5-Ag_2_O@ChNF ~2.40 mmol·g^−1^. Ag_2_O-free ChNF aerogels were also used to adsorb iodine anions as a control. Pristine aerogels removed 13.8% iodine anions in solution with an adsorption capacity of 0.41 mmol/g. Amine groups at deacetylated C2 positions in ChNFs may be attracted to and capture I^−^ via electrostatic interaction. Capture capacity, however, was not proportional to Ag_2_O content. This disproportionate relationship may be attributed to change in Ag_2_O particle size, specific surface area, and pore volume. When loading content increased from 31 wt% to 122 wt%, the specific surface area decreased from 131.96 m^2^/g to 74.31 m^2^/g and pore volume decreased from 0.66 cm^3^/g to 0.27 cm^3^/g. Larger Ag_2_O particles thickened the pore walls of ChNF aerogels and decreased specific surface area and pore volume. These effects collectively decreased the adsorption capacity of aerogels with high Ag_2_O content below those of aerogels of low Ag_2_O content. The absorbability of Ag_2_O@ChNF aerogels remains considerably higher than those of Hg- and Cu-based adsorbents: the maximum adsorption capacity of cinnabar is only 19 μmol/g and that of Cu_2_S is 48 μmol/g^29, 30^. The iodide adsorption capacity of several sorption materials are listed in Supplementary Table S1.

The high-efficiency adsorption capacity and fast adsorption kinetics of Ag_2_O@ChNF are dependent on the specific area of Ag_2_O particles. Nanosized Ag_2_O particles with large specific area are more active and have more opportunities for exposure to I^−^ than Ag_2_O agglomerates or large Ag_2_O particles. In addition, the large specific area and high porosity of ChNF aerogels provide fine Ag_2_O nanoparticles with ample space for uniform dispersion to avoid particle aggregation, thus accelerating adsorption.

We investigated the selectivity of Ag_2_O@ChNF aerogels for capturing I^−^ when I^−^ coexisted with Cl^−^. As shown in Fig. 5c, I^−^ adsorption decreased at high Cl^−^ concentrations. At a Cl^−^ and I^−^ molar ratio of 100, 88% I^−^ was removed. When Cl^−^ and I^−^ molar ratio increased to 1000, 48% I^−^ was removed. Although competitive adsorption occurred when Cl^−^ coexisted with I^−^, adsorption capacity still reached 1.59 mmol/g (Cl^−^: I^−^ = 1000), which is higher than those reported for Bi-based adsorbents (see Supplementary Table S1).

**Figure 6:**
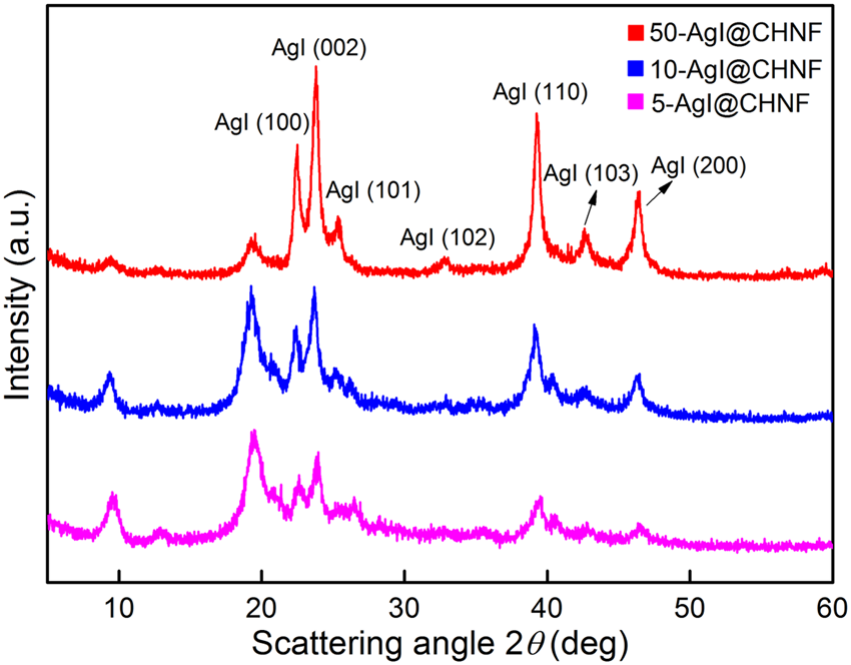
XRD patterns of 50- AgI@ChNF, 10-AgI@ChNF, and 5-AgI@ChNF.

During reaction with NaI solution, the formation of AgI@ChNF composite caused the color of Ag_2_O@ChNF aerogels to change to yellowish-white. Figure 6 shows the XRD patterns of Ag_2_O@ChNF samples with different loading concentrations after the adsorption test. The diffraction peak of hexagonal *β*-AgI appeared at 22.3°, 23.7°, 25.4°, 39.2°, 42.6°, and 46.3°, which corresponded to the (100), (002), (101), (110), (103), and (112) planes of *β*-AgI. By contrast, the previous diffraction peaks of Ag_2_O disappeared. The TEM images of AgI nanoparticles formed via I^−^ adsorption were collected (Supplementary Fig. S5).

**Figure 7:**
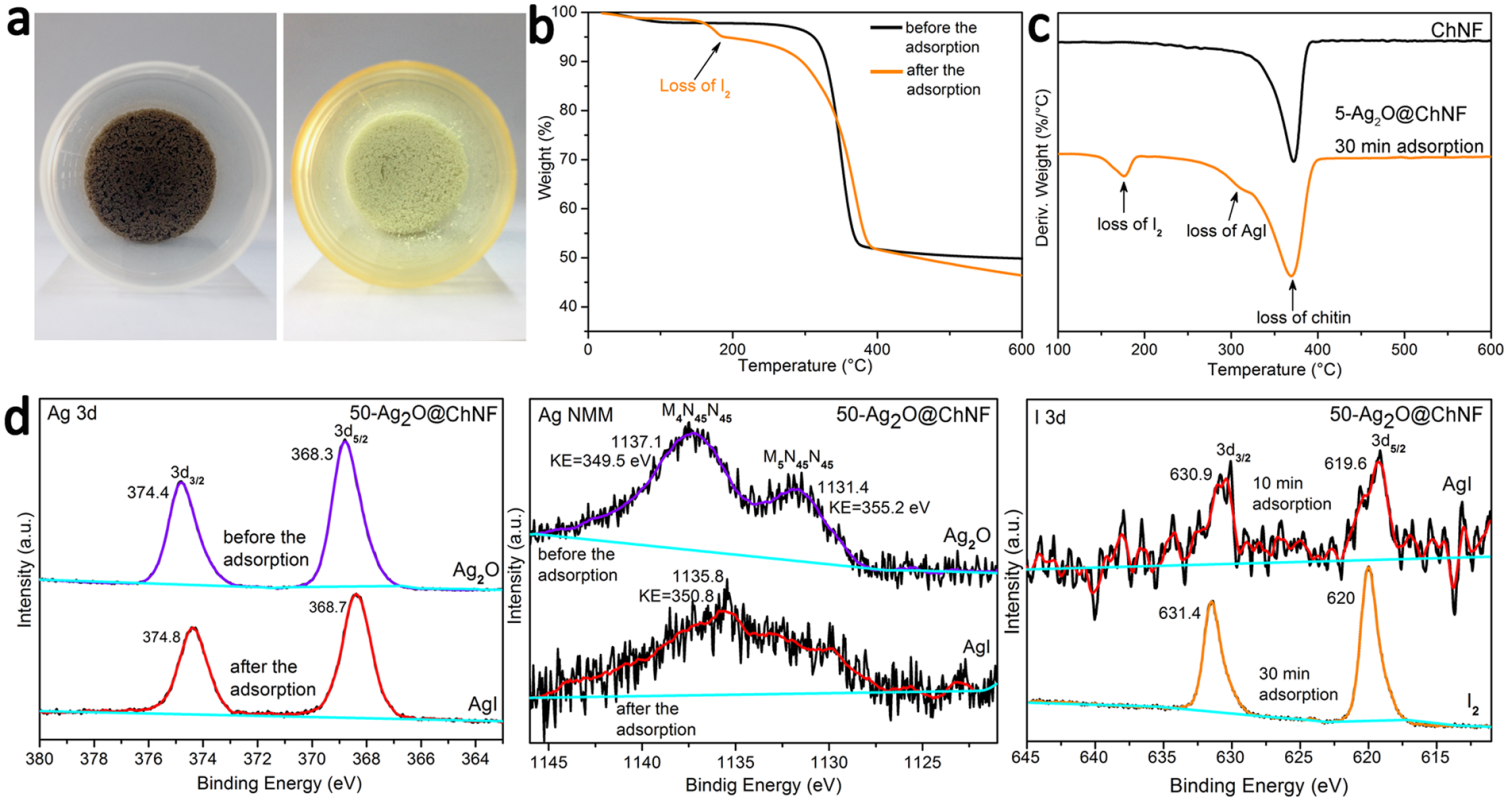
(**a**) Image of the aerogel before and after the I_2_ adsorption. (**b**) TG analysis of ChNF aerogel before and after I_2_ adsorption. (**c**) DTG analysis of pristine ChNF and AgI@ChNF of 5-Ag_2_O@ChNF, 30 min adsorption. (**d**) Ag 3d photoelectron spectra and Ag MNN Auger spectra of 50-Ag_2_O@ChNF before and after I_2_ capture. I 3d photoelectron spectra of 50-Ag_2_O@ChNF with adsorption time of 10 and 30 min.

To investigate the effects of ultrasonication on the agglomeration of nanoparticles, we did not treat some samples with ultrasonication prior to freeze-drying. AgI was formed as disordered agglomerates without a specific orientation. This result further proved the effectiveness of ultrasonication on the prevention of nanoparticle agglomeration. Agglomeration prevented AgI from detaching from the ChNF network. The cross-linked 3D ChNF network locked agglomerates in mesopores, preventing their leakage into the solution. During the capture test, Ag_2_O chemically transformed to AgI. On a macroscopic level, the color change of the aerogel indicated this chemical phase transformation. As show in Fig. 7(a), after exposure to iodine vapor, the color of Ag_2_O@ChNF turned from brownish-black to light yellow. Further investigation revealed that I^−^ anchored on the ChNF scaffolds not only in the form of AgI but also as molecules. This result was verified by thermoanalysis. The thermogravimetric (TG) analysis of pristine ChNF aerogel before and after the capture test is presented in Fig. 7(b). A broad mass loss step is observed from 155 °C to 190 °C (I_2_ bp = 184 °C), which was designated as the decomposition of I_2_. Moreover, a weight loss peak also appeared in a similar temperature range in the DTG spectra line of AgI@ChNF, as shown in Fig. 7(c). Therefore, AgI@ChNF aerogels can capture a portion of iodine vapor in the form of iodine molecules. In DTG analysis, as shown in Fig. 7(c), a major weight loss peak at ~380 °C appeared in the spectral lines of pristine ChNF and AgI@ChNF aerogels. This major weight loss peak indicated the decomposition of the chitin scaffold. An inapparent peak that appeared at ~318 °C represented the loss of AgI. The above results were further confirmed by the X-ray photoelectron spectroscopy (XPS) spectra of Ag 3d, as shown in Fig. 7(d). However, given that the shifts in the binding energies of chemical phase transformation in XPS line shapes for Ag are inconspicuous, Ag MNN Auger analysis was performed to help identify the chemical states of composite materials^31^. Before I_2_ adsorption, the binding energy of the Ag 3d_5/2_ photoemission peak was 368.3 eV. Ag MNN bimodal peaks were observed in the spectrum and the binding energy of Ag M_5_N_45_N_45_ was 355.2 eV, which confirmed that Ag_2_O particles anchored on the surfaces of the ChNF scaffold. After the adsorption test, a positive shift of binding energy to 0.4 eV was observed, the binding energy of Ag 3d_5/2_ in samples turned to 368.7 eV, and the doublet peak in the Auger spectrum disappeared. Moreover, the binding energy of Ag M_5_N_45_N_45_ was 350.1 eV. These results collectively provide evidence for the conversion of Ag_2_O to AgI. The XPS spectrum of I 3d was also investigated to verify the results of TG analysis. The I 3d_5/2_ binding energy of 50-Ag_2_O@ChNF at the adsorption time of 10 min was measured at 619.6 eV. After 30 min, the binding energy of I 3d_5/2_ was 620 eV, which corresponded to the binding energy of I_2_. The analytical results mentioned above indicated that Ag_2_O@ChNF rapidly and efficiently fixed I_2_ in the form of AgI. Moreover, by prolonging the duration of adsorption, molecular I_2_ can also be anchored inside the aerogels. Therefore, the high efficiency of Ag_2_O@ChNF heterostructured aerogel indicates its potential application in radioactive iodine adsorption.

In conclusion, a green and facile fabrication process was developed for the preparation of nanofibrillated Chitin/Ag_2_O heterostructured aerogels. ChNFs were first dissociated from purified chitin via a simple ultrasonication treatment. The ChNF building blocks provided abundant acetyl amino groups as active sides for interaction with Ag_2_O. Host materials were then obtained after the assembly of ChNFs into pristine monolithic aerogels. ChNF aerogel had an ultra-low density of 2.19 mg/cm^3^. The final products were obtained after immersion in Tollen’s reagent followed by freeze-drying. Ag_2_O@ChNF aerogels have characteristics that are advantageous for radioactive iodine capture. The physical entanglement of ChNFs provided the composited aerogels with a highly mesoporous structure and a high specific surface area. Fine nanosized Ag_2_O particles conferred the high efficiency of this material efficient for iodine immobilization. The adsorption capacity of Ag_2_O@ChNF aerogels for I^−^ anions reached up to 2.81 mmol·g^−1^. The results of the vapor adsorption test revealed that this monolithic composite material rapidly captured iodine vapor in the form of AgI and as molecular iodine. Therefore, this material, which was derived from green bio-resources, is an innovative alternative for radioactive iodine capture and has considerable potential applications in radioactive waste disposal.

## Methods

### Chemical purification of chitin

To eliminate lipids and pigments from the samples, 2 g carapace powders was submerged in a 2:1 (v:v) mixture of ethyl acetate and ethanol (95%) and purified using a Soxhlet apparatus at 90 °C for 6 h under reflux. The samples were rinsed with distilled water and then treated with 5 wt% KOH under strong agitation for 48 h to remove protein. The samples were cooled to room temperature, thoroughly washed with distilled water, and treated with 7 wt% hydrochloric acid under magnetic stirring at room temperature for an additional 48 h to remove mineral salts. The treated samples were then washed with distilled water and filtered. Next, the samples were treated with an acidified sodium chlorite solution (1.5 wt% NaClO_2_, buffered with acetic acid to pH 4.0) at 80 °C for 1 h. This process was repeated 6 times. After the above treatments, the purified chitin was immersed in distilled water to maintain moisture and to prevent strong hydrogen bonding between fibers during drying.

### Fabrication of chitin nanofibers (ChNFs)

A total of 0.5 wt% purified wet chitin was dispersed in 400 mL distilled water. The sample was sonicated at 20 kHz at the work cycle of 1 s with a YJ99-IIDN sonicator (Ningbo Scientz Technology Co., ltd) with a 1-cm^2^-diameter titanium horn and an output power of 1300 W. Sonication was performed in an ice-water bath. The ChNF solution was obtained after sonication.

### Synthesis of ChNF aerogels

The ChNF suspension was loaded into dialysis bags, which were then subjected to solvent-exchange with *tert*-butanol. After this process, the low-viscosity ChNF suspension first turned into a diaphanous, pulpy substance, and finally to a gel body. The obtained gel was divided equally and poured into molds. The samples were then freeze-dried at −55 °C for 12 h at 25 μpa *in vacuo*, finally yielding ChNF aerogels.

### Synthesis of Ag_2_O@ChNF aerogels

ChNF aerogels were immersed in freshly prepared Tollen’s reagent at a molar ratio of [Ag]/[NH_3_] = 1:2. Samples were then treated via ultrasonication for 5 min to ensure that particles were homogeneously dispersed in the ChNF network. Samples were prepared with 5 × 10^−2^, 1 × 10^−2^, or 5 × 10^−3^ mol/L Ag(NH_3_)_2_OH; transferred to NaOH solution (pH = 13); and allowed to stand in open containers for 12 h at room temperature for the full reaction. The colors of samples gradually changed from white to brownish black as nanoparticles formed. Nanoparticle-loaded ChNF networks were then washed with distilled water thrice to remove heteroions. Composited networks were frozen at −30 °C and freeze-dried to obtain monolithic aerogels with fine Ag_2_O fine granules anchored on ChNF networks. Final products prepared with 5 × 10^−2^, 1 × 10^−2^, or 5 × 10^−3^ mol/L Ag(NH_3_)_2_OH were denoted as 50-Ag_2_O@ChNF, 10-Ag_2_O@ChNF, or 5-Ag_2_O@ChNF, respectively.

### I^−^ adsorption

For safety concerns, we used the nonradioactive I isotope to monitor the behavior of ^131^I^−^ and ^129^I^−^. Ag_2_O@ChNF adsorbents with different loading concentration were added to a series of aqueous NaI solutions at concentrations of 5 to 25 ppm. Per milligram absorbents was assigned to 15 mL of NaI solution. An appropriate amount of reaction solution was sampled and centrifuged at specific time intervals. The supernatant was used to measure the adsorption spectra of I^−^ via UV–Vis measurement. The kinetic isotherms of I^−^ adsorption by Ag_2_O@ChNF samples were determined at 20 ppm with a piece of pristine aerogel as the control. The complete color conversion of the adsorbents indicated the completion of the reaction. Adsorbents were recovered and dried for further characterization.

### Selective uptake of I^−^ anions by Ag_2_O@ChNF aerogels

The selective uptake of I^−^ was investigated in the presence of high concentrations of Cl^−^ anions. Per milligram absorbents (50-Ag_2_O@ChNF) was assigned to a 15-mL mixture solution of NaI and NaCl, where NaI concentration was 20 ppm and the molar ratios of Cl^−^ to I^−^ were set to 100:1 and 1000:1, respectively. The solution was shaken for 45 h. At specific time intervals, an appropriate amount of solution was sampled and centrifuged. The supernatant was subjected to UV–vis measurement.

### Capture of iodine

A 500 mg sample of I_2_ was placed in a 50-mL beaker. Then, a piece of fritted glassware with a piece of Ag_2_O@ChNF gel was placed on the beaker. The apparatus was heated to 75 °C to evaporate iodine. During this process, a distinct color distribution appeared on the aerogels. The complete color change of the aerogel sample indicated that the reaction was completed. A piece of ChNF aerogel was also used to capture iodine vapor. Materials after I^−^ adsorption and I_2_ vapor capture were denoted as AgI@ChNF.

### Characterization

The ^13^C NMR experiments were performed on a 500 MHz nuclear magnetic resonance spectorometer (Bruker advance III HD 500 MHz). The bulk density of ChNF aerogels was determined on the basis of the physical dimensions and weights of the samples. The microstructures of ChNF and monolithic aerogels were observed via SEM (Sirion 200, FEI). Samples were coated with gold in advance to improve conductivity. Adsorption–desorption isotherm measurements were obtained with a JW-BK132F specific surface area and pore size analyzer. Specific surface area was calculated in accordance with the Brunauer–Emmett–Teller method. Pore size distribution was estimated via BJH method. The TEM analysis of the samples was performed on a FEI, Tecnai G2 F20 TEM at 200 kV. The appropriate amount of Ag_2_O@ChNF was dispersed in alcohol and dispersed with an ultrasonic cleaner. One microliter of suspension was taken out by pipette and dropped on the support film and air dried for TEM observation. The XRD patterns of the Ag_2_O@ChNF samples before and after adsorption of I^−^ were measured with D/max 2200, Rigaku powder XRD instrument using Ni-filtered Cu Kα radiation (*λ* = 1.5406 Å). Data were collected over a 2*θ* from 5° to 60° at a scanning rate of 4°/min. FTIR spectra were recorded on a Nicolet Nexus 670 FTIR instrument in the range of 500–4000 cm^−1^ at a resolution of 4 cm^−1^. I^−^ concentration was measured and calculated by a Shimadzu UV-2501 PC spectrometer. Thermogravimetric analysis (TGA) was performed in a Shimadzu TGA-50 thermal analyzer by heating each sample from room temperature to 800 °C with a ramping rate of 5 °C min^−1^ under nitrogen flow. The composition of the composite samples was investigated via X-ray photoelectron spectroscopy using a Thermo Escalab 250 Xi XPS spectrometer. The C1s peak of adventitious carbon at 285 eV was used as a binding energy reference.

## Electronic supplementary material

Supplementary Information
