# Supplementary material for: Facile Fabrication of Nanofibrillated Chitin/Ag2O Heterostructured Aerogels with High Iodine Capture Efficiency
Source: Sci Rep. 2017 Jun 27;7:4303. doi: 10.1038/s41598-017-04436-8 (PMC5487321; doi:10.1038/s41598-017-04436-8)
Supplement: Supplementary file 1 — Supplementary Information [file 41598_2017_4436_MOESM1_ESM.pdf]

Supplementary Information

Facile Fabrication of Nanofibrillated Chitin/Ag_2O Heterostructure Aerogels with High Iodine Capture Efficiency

Runan Gao[1,3,+], *Yun Lu*[2,3,*,+], *Shaoliang Xiao*[1,3] & *Jian Li*[1,3,*]

[1] Material Science and Engineering College, Northeast Forestry University

Harbin, 150040 (P.R. China)

[2] Research Institute of Wood Industry, Chinese Academy of Forestry

Beijing, 100091, (P.R. China)

[3] Key Laboratory of Bio-based Material Science and Technology Ministry of Education, Northeast Forestry University

Harbin, 150040 (P.R. China)

[*] Corresponding author.

E-mail address: jianlinefu@126.com

The deacetylation degree (DD) of chitin was evaluated from subtract the relative integral of methyl groups compared to the carbon integrals of the polysaccharidic backbone from one.[1] The DD was calculated by:

$$DD = 1 - \frac{100 \times I_{N-CH_3}}{\frac{1}{6} \times \left(I_{C1} + I_{C2} + I_{C3} + I_{C4} + I_{C5} + I_{C6} \right)} \tag{1}$$

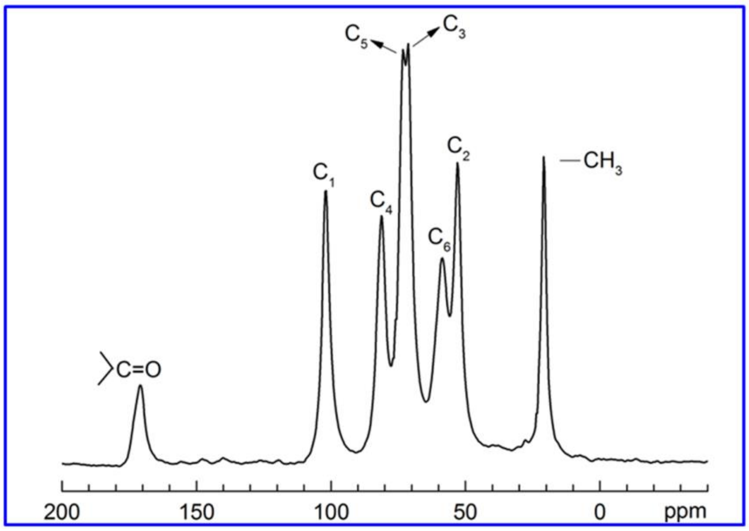

Figure S1. ^{13}C NMR spectra of ChNFs after chemical pretreatment.

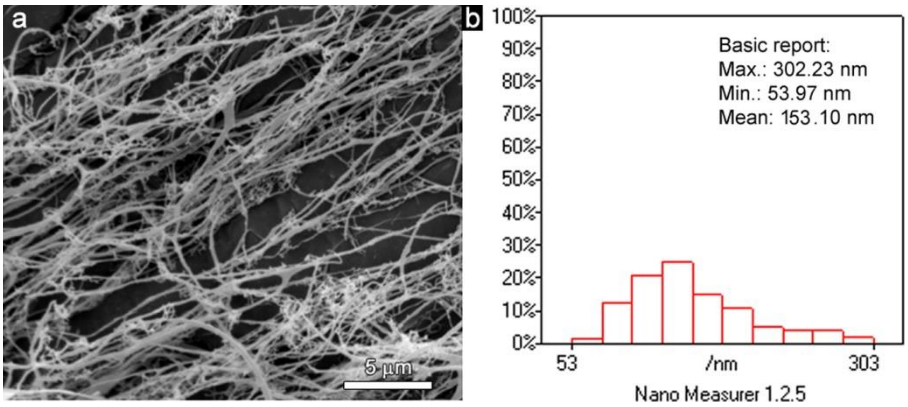

Figure S2. (a) ChNFs obtained after 30 min ultrasonication treatment (a) and corresponding diameter distribution report (b).

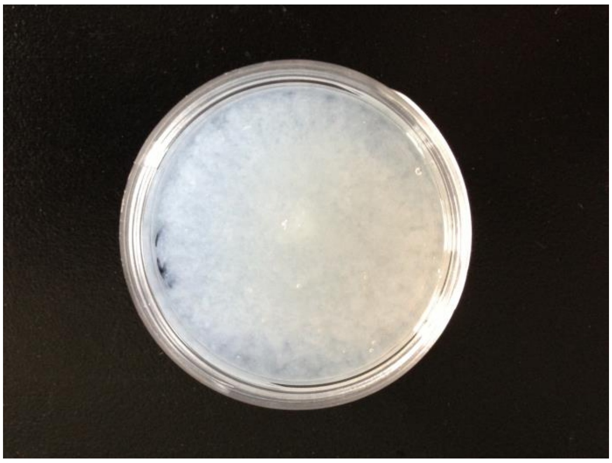

Figure S3. The image of ChNFs after the solvent-exchange with *tert*-butanol.

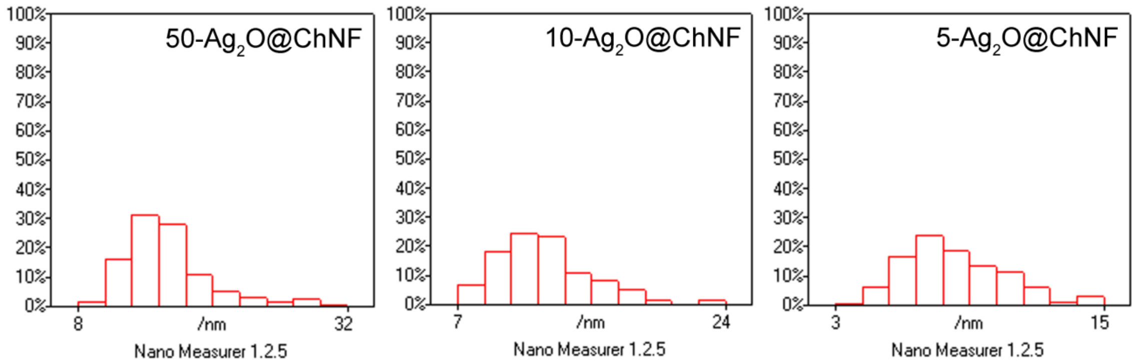

Figure S4. The size distribution of Ag_2O nanoparticles of Ag_2O@ChNF with decreasing loading concentrations.

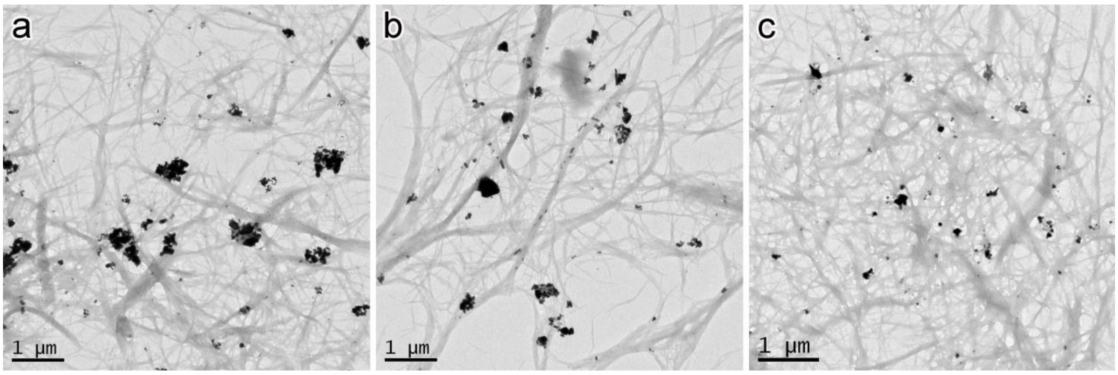

Figure S5. TEM images of AgI@ChNF samples (**a**) 50- AgI@ChNF, (**b**) 10-AgI@ChNF and (**c**) 5- AgI@ChNF after I- anions adsorption tests.

Table S1. Comparison of several iodide sorption materials.

Materials	Adsorption capacity (mmol/g)	Equilibrium time (h)	Selectivity adsorption (mmol/g)	Ref.
HgS	0.005	—	—	2
Cu_2S	0.002	120	—	3
Cu_2O	0.048	336	—	4
α-Bi_2O_3	0.70	10	0.44	5
MR-δ-Bi_2O_3	1.44	0.25	1.38	6

Table S2. Crystallite size of Ag_2O nanoparticles measured from Scherrer equation and TEM observation.

Samples	Crystallite size (nm)	
	Scherrer equation	TEM
5-Ag_2O@ChNF	9	8
10-Ag_2O@ChNF	12	13
50-Ag_2O@ChNF	14	16

References

1. L. Heux, †, J. Brugnerotto, J. Desbrières, M.F. Versali, a. & Rinaudo†, M. Solid State NMR for Determination of Degree of Acetylation of Chitin and Chitosan. *Biomacromolecules* **1**, 746 (2000).
2. Sazarashi, M. Adsorption of I Ions on Cinnabar for 129I Waste Management : Radiochimica Acta. *Radiochimica Acta* **65**, 195-198 (1994).
3. Lefèvre, G., Bessière, J., Ehrhardt, J. J. & Walcarius, A. Immobilization of iodide on copper(I) sulfide minerals. *Journal of Environmental Radioactivity* **70**, 73 (2003).
4. Lefèvre, G., Walcarius, A., J.J. Ehrhardt, a. & Bessière, J. Sorption of Iodide on Cuprite (Cu2O). *Langmuir* **16**, 4519-4527 (2000).
5. Kodama, H. Solidification of Iodide Ion by Reaction with Bi2O3. *Bulletin of the Chemical Society of Japan* **65**, 3011-3014 (1992).
6. Liu, L. *et al.* Selective Capture of Iodide from Solutions by Microrosette-like δ-Bi2O3. *Acs Applied Materials & Interfaces* **6**, 16082-16090 (2014).
